# Use of quantitative CT chest imaging to derive and assess a radiographic phenotype of deployment-related constrictive bronchiolitis

**DOI:** 10.1186/s12931-025-03269-8

**Published:** 2025-05-21

**Authors:** Alexander J. Bell, Maria Masotti, Sundaresh Ram, Gregory Pappas, Robert F. Miller, Ella A. Kazerooni, Charles R. Hatt, MeiLan K. Han, Bradley W. Richmond, Michael J. Falvo, Craig J. Galban, John J. Osterholzer

**Affiliations:** 1 Department of Radiology, University of Michigan, Ann Arbor, MI USA; 2 Department of Biostatistics, University of Michigan, Ann Arbor, MI USA; 3 Department of Radiology and Imaging Sciences, Emory University, Atlanta, GA USA; 4 Wallace H. Coulter Department of Biomedical Engineering, Emory University, Georgia Institute of Technology, Atlanta, GA USA; 5 Airborne Hazards and Burn Pits Center of Excellence, VA New Jersey Health System, East Orange, NJ USA; 6 Division of Allergy, Pulmonary, and Critical Care Medicine, Vanderbilt University Medical Center, Nashville, TN USA; 7 Division of Pulmonary and Critical Care Medicine, University of Michigan, 2215 Fuller Rd (111G), Ann Arbor, MI 48105 USA; 8 Department of Veterans Affairs Medical Center, Nashville, TN USA; 9 New Jersey Medical School, Rutgers University, Newark, NJ USA; 10 Pulmonary Section, VA Ann Arbor Health System, Ann Arbor, MI USA

**Keywords:** Phenotypes, Airway disease, Environmental exposures, Military deployment, Lung injury

## Abstract

**Background:**

Efforts to phenotype veterans that developed respiratory symptoms following deployments to the Southwest Asia Theater of Military Operation have been limited by the insensitivity of current non-invasive testing to objectively identify deployment-related constrictive bronchiolitis and other features of chronic lung injury. In this study, we derived a quantitative CT (QCT)-based radiographic phenotype of biopsy-proven deployment-related constrictive bronchiolitis (DRCB) and assessed its ability to assist in the phenotyping of non-biopsied formerly deployed symptomatic veterans.

**Methods:**

QCT analysis combined with demographic, physiologic, symptom, and exposure data was obtained from three cohorts: military personnel with biopsy-proven deployment-related constrictive bronchiolitis (DRCB, *n* = 37), formerly deployed symptomatic veterans (FDSV, *n* = 71), and asymptomatic civilians (Control, *n* = 98). Differences in unadjusted QCT metrics and demographic variables between cohorts were identified and further assessed by principal component analysis. Thereafter, adjusted data from the DRCB cohort was used to derive a QCT-based radiographic phenotype of DRCB expressed as a DRCB-Probability Index (DRCB-PI). Application of the DRCB-PI to the FDSV cohort was used to assess additional phenotypic metrics associated with the DRCB phenotype (DRCB-PI > 0.5).

**Results:**

Individual unadjusted QCT metrics for functional small airways disease and high attenuation area were elevated in DRCB and FDSV cohorts (relative to Control). Primary component analysis revealed that DRCB and FDSV cohorts overlapped and were distinguished from the Control cohort. The FDSV subjects whose DRCB-PI was > 0.5 had greater evidence of small airways disease (assessed by oscillometry and QCT) and self-reported more intense immediate health effects to their exposures to military burn pit smoke, and sand and dust.

**Conclusions:**

Application of a QCT-derived radiographic phenotype of DRCB identified a subset of veterans with evidence of abnormal small airways and more severe self-reported health effects following inhalational exposures during military deployment. Future studies incorporating QCT may help establish non-invasive strategies to detect DRCB and other forms of chronic lung injury.

**Supplementary Information:**

The online version contains supplementary material available at 10.1186/s12931-025-03269-8.

## Introduction

Chronic respiratory symptoms are commonly reported among the approximately 3.7 million U.S. military personnel deployed to the Southwest Asia Theater of Military Operations 1, 2, 3, 4. A recent study 5 identified associations between exposures to burn pit smoke and military occupational vapors, gases, dust, and fumes with increased chronic respiratory symptoms. Additionally, asthma, COPD, and hypertension (defined by diagnostic codes) were more frequent amongst military personnel deployed to bases with open air burn pits 6. However, diagnoses are often elusive in as many as one-third of individuals despite a thorough non-invasive evaluation 7.

Constrictive bronchiolitis (CB) has been identified in a subset of formerly deployed military personnel that underwent surgical lung biopsy following an inconclusive non-invasive evaluation for chronic respiratory symptoms 8, 9. In addition to CB, subsequent studies have identified additional histopathologic abnormalities in many subjects suggestive of multi-compartmental lung injury including findings of chronic airway inflammation, interstitial abnormalities, airspace enlargement, and vascular remodeling 9, 10, 11. Unfortunately, non-invasive diagnostic approaches for deployment-related CB (DRCB) are difficult owing to the insensitivity of conventional pulmonary function tests (PFTs) and reliance on qualitative impressions of high-resolution CT (HRCT) scans 8, 9. This has prompted calls to develop new non-invasive methods to identify and phenotype DRCB and related patterns of chronic lung injury 1, 12.

Parametric response mapping (PRM) has emerged as an effective form of quantitative CT (QCT) analysis that quantifies multiple radiographic abnormalities, including features of functional small airways disease (fSAD), by measuring differences in lung density obtained from spatially aligned inspiratory and expiratory HRCT images 13, 14, 15, 16, 17. Using PRM, we previously demonstrated that %PRM^fSAD^ in military personnel with biopsy-proven DRCB is increased relative to asymptomatic control subjects and subjects with spirometric evidence of mild to moderate COPD 18. This finding identified %PRM^fSAD^ as a potential QCT indicator of DRCB, yet a more complete QCT phenotype of DRCB using multiple PRM metrics has not been assessed.

In the current study, we performed PRM analysis on HRCT scans obtained from subjects with biopsy proven DRCB, non-biopsied formerly deployed symptomatic veterans (FDSV), and asymptomatic control subjects to identify QCT metrics associated with biopsy-proven DRCB. We then derived a radiographic phenotype of DRCB and assessed its ability to enhance the phenotyping of the FDSV cohort when incorporated with additional metrics of pulmonary function, airway wall thickness and ventilation, symptoms, and inhalational exposures.

## Methods

### Study populations

Three separate cohorts of subjects were used in this study: (1) military personnel with DRCB evaluated at Vanderbilt Medical Center [DRCB, *n* = 37, detailed in 8, 18)], (2) formerly deployed symptomatic veterans evaluated at the New Jersey VA War Related Illness and Injury Study Center (FDSV, *n* = 71), and (3) asymptomatic non-military adults from A Study to Obtain Normal Values of Inflammatory Variables from Healthy Subjects [Control, *n* = 98, detailed in: 18, 19]. All subjects in the Control cohort consented to participate. Consent to participate was waived (following IRB review) for the DRCB and FDSV cohorts since their data were obtained for clinical indications. The study was conducted in accordance with approvals obtained following IRB review at the VA Ann Arbor Health System (1754681), the University of Michigan (HUM00246250), the Vanderbilt Medical Center, and the VA New Jersey Health System. Additional descriptions of the cohorts are provided below and in the Supplemental Material.

### Assessments

Demographic variables including age, gender, tobacco use history, and body mass index (BMI) were recorded for all subjects.

All subjects underwent complete pulmonary function testing (PFT), including spirometry (FEV_1_, FEV_1_/FVC), body plethysmography (TLC, RV/TLC), and diffusing capacity of carbon monoxide (DLCO) according to American Thoracic Society guidelines 20, 21, 22 with results reported as a percent predicted 23, 24, 25 (see Supplemental Material). The FDSV subjects also underwent respiratory oscillometry (R5, R5-R20, X5, AX) performed in accordance with standard guidelines 26 as reported previously 27 and further detailed in the Supplemental Material.

Volumetric HRCT scans were obtained at full inspiration and at approximate residual volume for all subjects. Scanner type and protocols are detailed in the Online Supplemental Material (e– Table 1).

The FDSV subjects were asked to self-report the intensity of health effects in response to smoke from burn pits, sand and dust, and petrochemicals during their deployment(s) that were graded on a scale from 1 to 4 as follows: (1) no noticeable health effects, (2) mild effects or symptoms that did not affect ability to conduct physical activities, (3) moderate effects or symptoms that had some effect on physical activity, or (4) severe effects or symptoms that markedly impaired physical activity and/or required medical treatment. Subjects also indicated the presence or absence of current cough, wheeze, and/or shortness of breath.

### QCT analysis

Parametric response mapping was applied to all paired HRCT scans as previously described 18, 28 and further detailed in the Online Supplemental Material. Each voxel, consisting of Hounsfield units at inspiration and expiration, were classified based on a scheme of predetermined thresholds 14 and Table 1 as: normal (PRM^Norm^), functional small airways disease (PRM^fSAD^), emphysema (PRM^Emph^), and high attenuation area (PRM^HAA^). The relative lung volumes, calculated as the sum of all voxels within a class normalized to the sum of all voxels within the expiratory lungs multiplied by 100, were used as global measures. QCT measurements of airway wall area, pi10 (wall thickness of airways with 10 mm internal perimeter), and Jacobian Mean (a measure of total lung ventilation) were performed as previously described 29, 30.

**Table 1:** Thresholds for parametric response mapping

	PRM^Norm^	PRM^fSAD^	PRM^HAA^	PRM^Emph^
Inspiration	-950 ≤ to < -810 HU	-950 ≤ to < -810 HU	-810 ≤ to < -250 HU	-1000 ≤ to < -950 HU
Expiration	-856 ≤ to < -250 HU	-1000 ≤ to < -856 HU	-1000 ≤ to < -250 HU	-1000 ≤ to < -856 HU

* HU, Hounsfield units

### Statistical analysis

Statistical analysis was performed using R Version 4.3.1. Unadjusted comparisons between cohorts were performed with a Kruskal Wallis test or Wilcoxon Rank-Sum test for continuous variables and a Chi-Square test or Fisher Exact test for categorical variables.

To derive a radiographic phenotype of DRCB using PRM metrics that accounted for potential confounders (including BMI, age, gender, and smoking status) we first fit a linear regression model in the Control cohort to obtain log-transformed PRM variables using BMI, age, gender, and smoking status (binary indicating ever smoker) as predictors. Using these fitted models (one for each PRM variable), we predicted the PRM values for each member of the three cohorts. We calculated the adjusted PRM values by subtracting the predicted PRM from the observed PRM values. The adjusted PRM variables represented the amount by which the PRM is elevated with respect to Control for a subject’s covariate profile. We then used the adjusted PRM variables to build a logistic regression model using logit link function 31 in the Control and DRCB cohorts. Cohort assignment was the dependent variable and adjusted HAA, adjusted fSAD, and their interaction were the independent variables. We constructed a receiver-operating characteristics (ROC) curve from this model and calculated the area under the curve (AUC). Finally, we applied the logistic regression model to the adjusted PRM variables of the FDSV cohort. We calculated a probability index of being DRCB (DRCB-PI) for each member of the FDSV cohort based on their adjusted PRM. To create binary sub cohorts for statistical analysis, we grouped the FDSV cohort into two groups: DRCB-PI > 0.5 (*n* = 10) and ≤ 0.5 (*n* = 61). We compared phenotypic metrics between the two groups using Fisher’s exact test for categorical variables or Wilcoxon rank sum test for continuous variables.

## Results

### Characteristics of the study populations

Baseline demographics and lung function for all cohorts are reported in Table 2. Overall, FDSV subjects were significantly older than DRCB (*P* < 0.001) and Control (*P* < 0.05) subjects. Similarities in median BMI and proportion of males were seen in the FDSV subjects when compared to DRCB subjects but found to be statistically higher than Control subjects (*P* < 0.001 and *P* < 0.001, respectively). The percentage of subjects who ever smoked were comparable between FDSV and DRCB subjects, yet less than Control subjects. The median pack years smoked for all FDSV subjects was comparable to Control subjects; this data was not available for the DRCB subjects. Median spirometry and lung volume measurements in all three cohorts did not identify obstructive or restrictive lung abnormalities. Despite all cohorts demonstrating no spirometric evidence of airflow limitation (FEV1/FVC > 0.7 as defined by GOLD), FDSV and DRCB subjects had significantly lower values of FEV1% predicted (pp) than Control subjects. The FDSV subjects did show significantly lower TLCpp values than observed from DRCB (*P* < 0.001) and Control subjects (*P* < 0.001). With respect to RV/TLC, a spirometric measure of air trapping, the ratio for FDSV subjects was similar to Control subjects and significantly lower than DRCB subjects (*P* < 0.01). No differences in DLCOpp were observed between cohorts.

**Table 2:** Study cohorts’ demographics and pulmonary function test results

	DRCB	FDSV	Control	*p*
**N**	37	71	98	
**Age (yrs)**	35 (30, 39)	45 (37, 52)	40 (22, 53)	< 0.001
**Sex (Male)**	34 (92%)	61 (86%)	55 (56%)	< 0.001
**BMI (Kg/m** ^**2**^ **)**	29.9 (28.4, 31.9)	31.7 (29.1, 36.0)	23.5 (21.4, 25.5)	< 0.001
**Ever-smoker (Y)**	13 (35%)	30 (42%)	55 (56%)	0.05
**Pack Years (yrs)**	-*	0 (0, 7)	1 (0, 16)	0.05
**FEV1pp (%)**	98 (86, 103)	95 (83, 107)	108 (101, 114)	< 0.001
**FEV1/FVC (%)**	81 (75, 85)	78 (75, 82)	79 (74, 83)	0.2
**TLCpp (%)**	100 (93, 107)	88 (80, 97)	105 (99, 108)	< 0.001
**RV/TLC (%)**	30 (26, 33)	25 (19, 30)	26 (23, 31)	0.005
**DLCOpp (%)**	95 (87, 101)	89 (78, 99)	90 (83, 99)	0.13

* Cohort missing adequate numerical measurements

Data are shown as median (lower quartile, upper quartile), or N (%)

### Comparison of QCT metrics using parametric response mapping

**Fig. 1:**
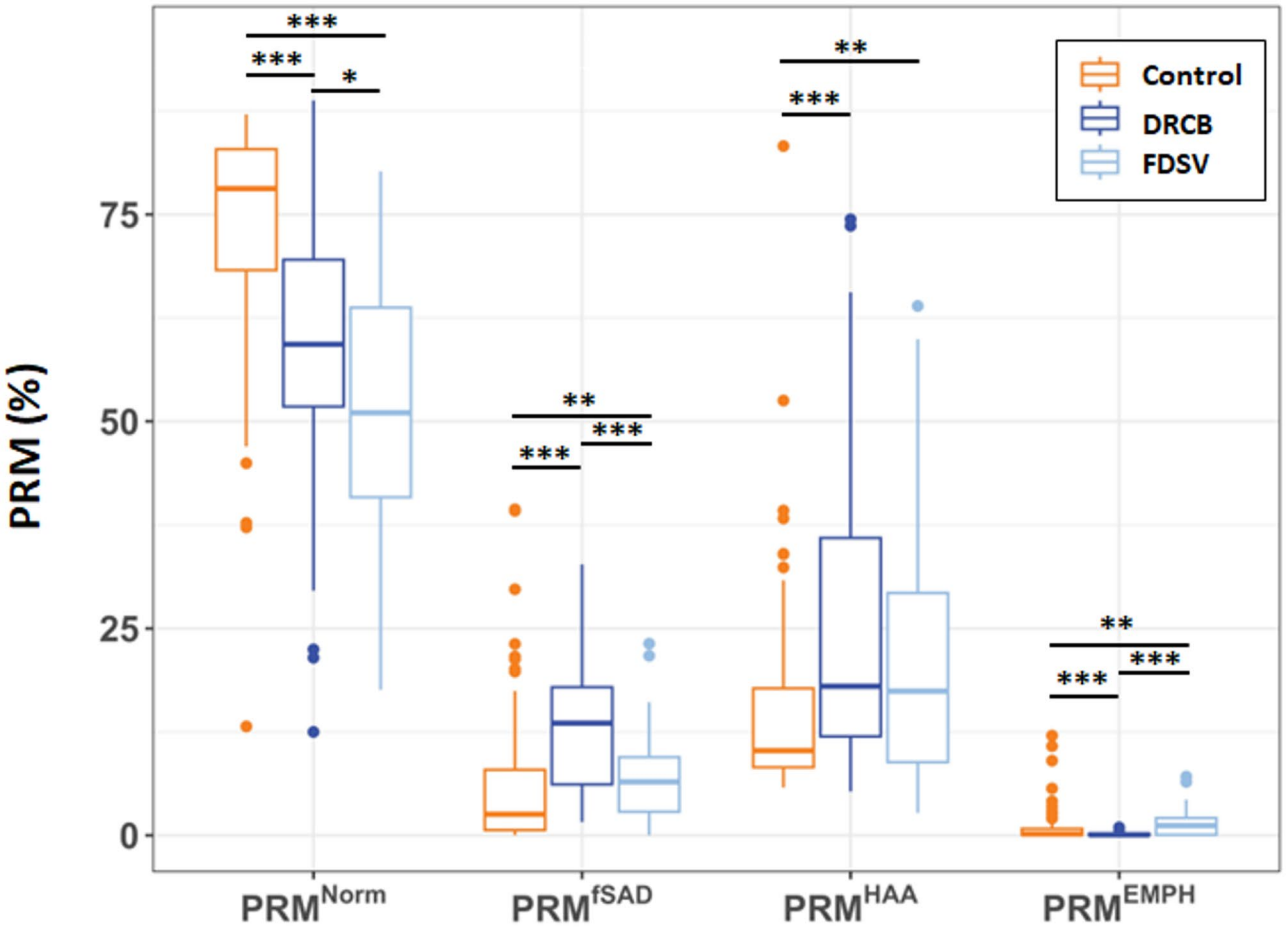
PRM metrics of DRCB, FDSV, and control subjects. The unadjusted analyses compared relative lung parenchymal volume involved in all 4 PRM metrics including normal (%PRM^Norm^), functional small airways disease (%PRM^fSAD^), high attenuation area (%PRM^HAA^), and emphysema (%PRM^Emph^). In the box plot, the horizontal line within the box represents the median and the top and bottom represent the upper and lower quartiles. The whiskers represent 1.5 times the interquartile range. Points outside the box and whiskers represent outliers. * = *p* ≤ 0.05, ** = *p* ≤ 0.01 and *** = *p* ≤ 0.001 by Wilcoxon Sum Rank test

A comparison of unadjusted PRM metrics between cohorts (Fig. 1) revealed that the median %PRM^Norm^ in the FDSV subjects was lower than that of the DRCB (*P* < 0.05) and Control (*p* < 0.001) subjects. The median %PRM^fSAD^ in the FDSV subjects was significantly less than the DRCB subjects (*P* < 0.001) but higher than Control subjects (*P* < 0.01) The median values of %PRM^HAA^ were comparable between FDSV and DRCB subjects and elevated relative to Control subjects. The median %PRM^Emph^ in the FDSV subjects was generally low yet increased relative to Control subjects (*P* < 0.01) and DRCB subjects (*P* < 0.001).

**Fig. 2:**
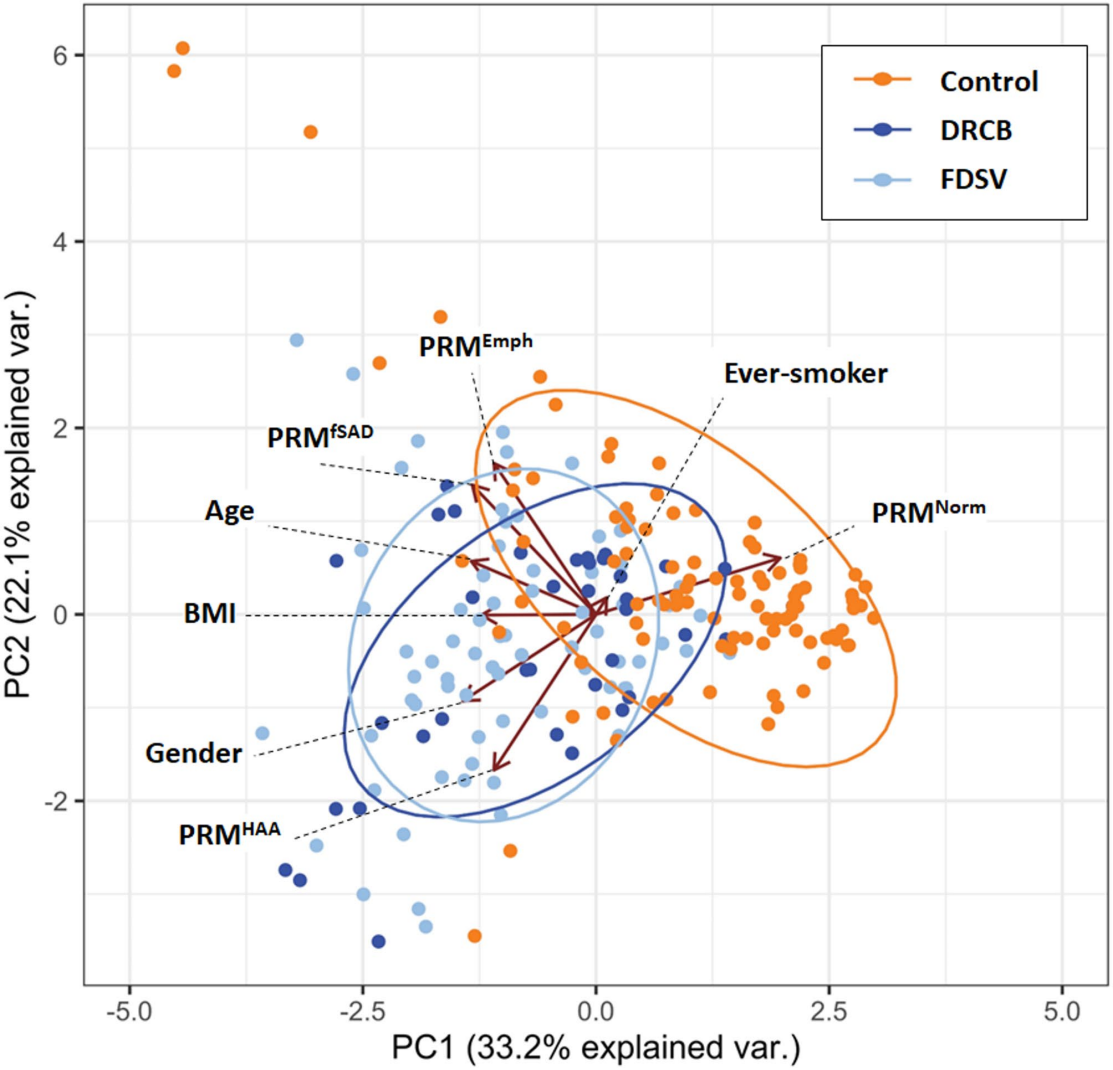
The PCA comparison between cohorts performed using all 4 PRM metrics and age, gender, BMI, and smoking status (ever/never). Error ellipses are plotted for each group with 60% confidence, and vectors are plotted from the origin using the loadings of the first and second principal component for the labelled features

To further compare these cohorts, a principal component analysis (PCA) was performed using all four PRM metrics, age, gender, BMI, and ever smoking status. Results demonstrate that variance in the first principal component is attributable primarily to differences in %PRM^Norm^ ($\:\iota\:$ = 0.54) with competing metrics being age ($\:\iota\:$ = -0.37), BMI ($\:\iota\:$ = -0.34), and gender ($\:\iota\:$ = -0.39), where $\:\iota\:$ is the loading coefficient. %PRM^fSAD^ ($\:\iota\:$ = 0.46), %PRM^Emph^ ($\:\iota\:$ = 0.54), and %PRM^HAA^ ($\:\iota\:$ = -0.57) contributed primarily to variance in the second principal component. Ever Smoking status was a minor factor in both cases. The resultant PCA plot (Fig. 2) shows that the FDSV subjects have a composite PRM phenotype that overlaps more closely with DRCB subjects relative to Control subjects.

### Derivation and assessment of a QCT-Based radiographic phenotype of DRCB

**Fig. 3:**
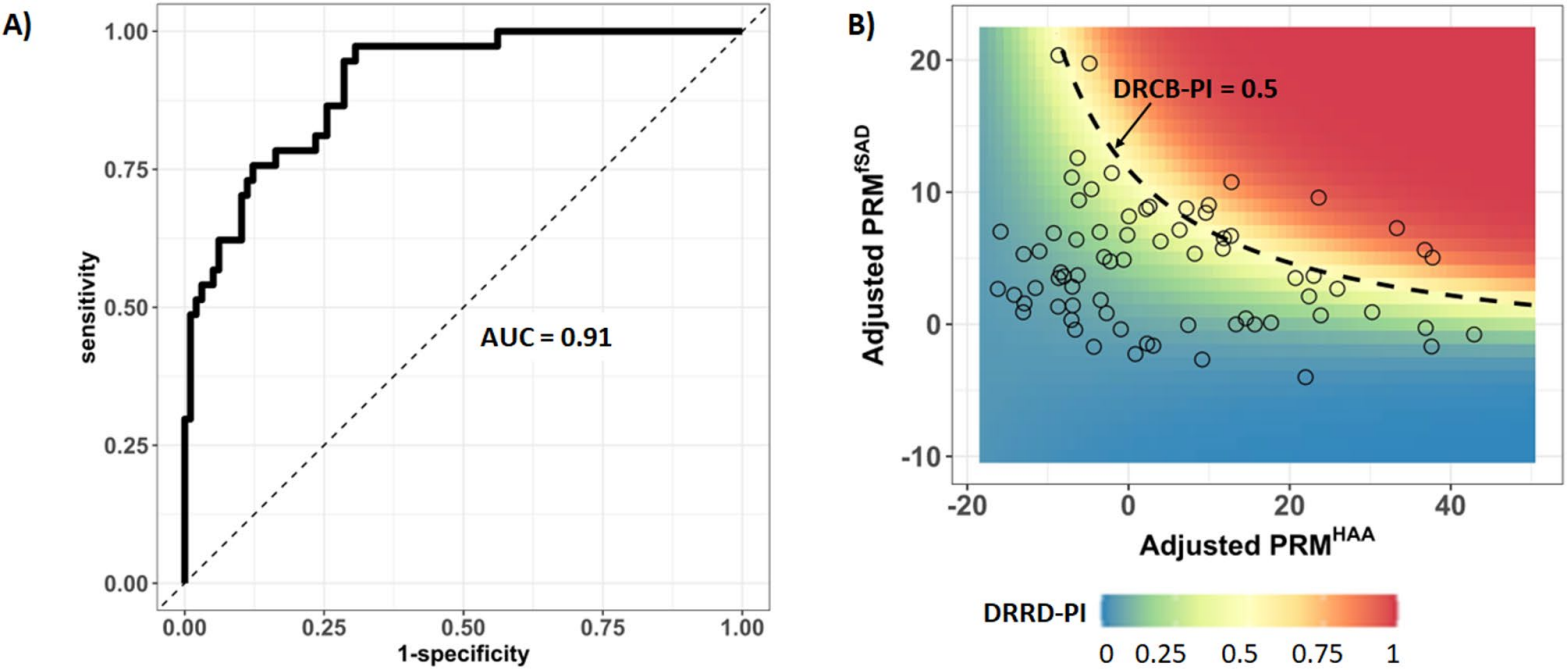
Identification of subjects with a radiographic phenotype of DRCB. (**A**) The area under the curve (AUC) plot derived from the model used to develop the DRCB-PI as an assessment tool for the identification of subjects with a radiographic phenotype of DRCB. (**B**) The application of the DRCB-PI to a cohort of 71 FDSV subjects (open circles) identifies 10 subjects with a DRCB-PI > 0.50 (dashed line). Colors indicate the probability of DRCB from 0 (dark blue) to 1 (dark red)

**Fig. 4:**
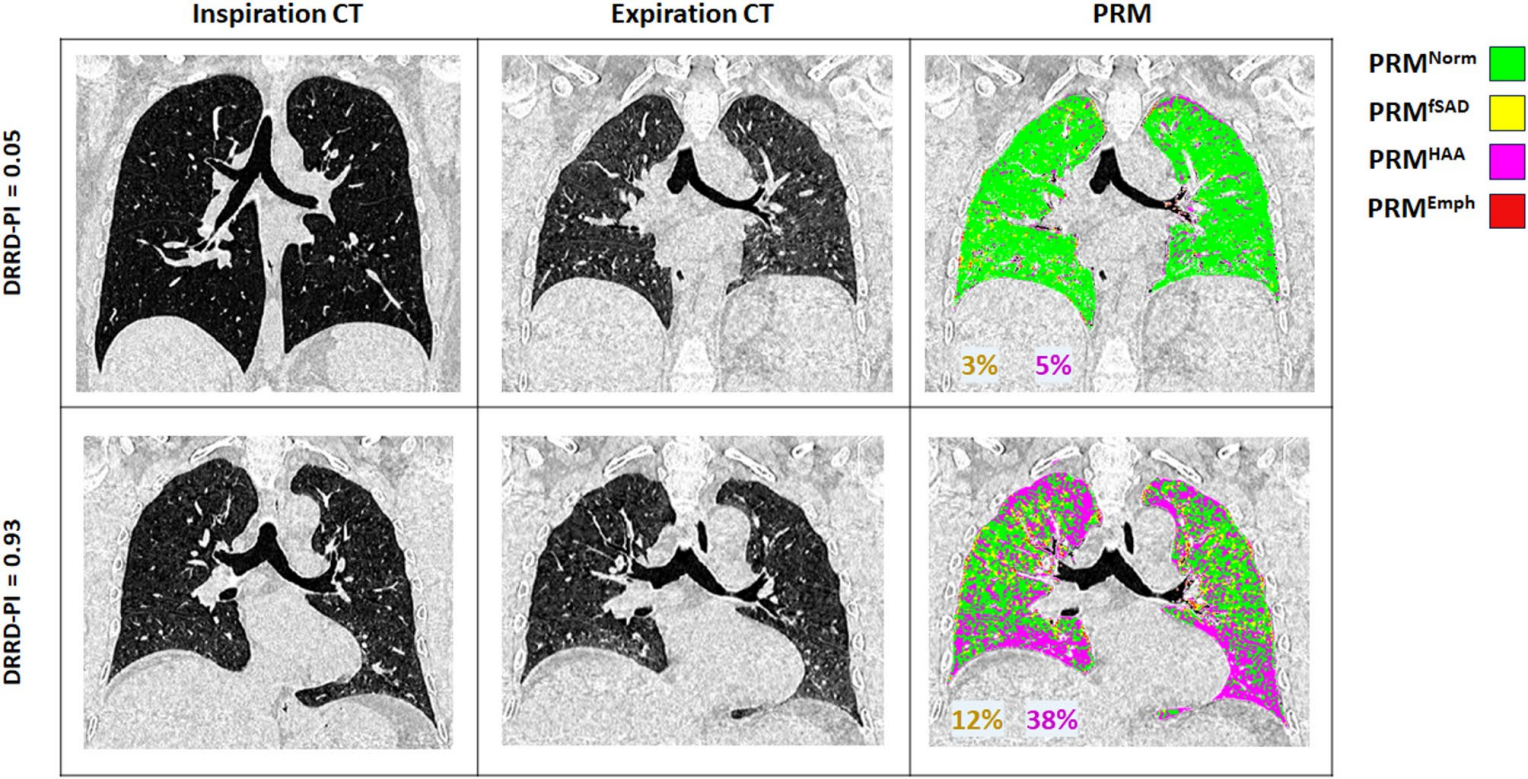
Representative HRCT images from FDSV subjects. Inspiratory (left panels), expiratory (middle panels), and PRM (right panels) images from FDSV subjects with a DRCB-PI < 0.5 (top row) and a DRCB-PI > 0.5 (bottom row). PRM images are color coded to identify lung tissue as normal (green), functional small airways disease (yellow), high attenuation area (pink), and emphysema (red). Percentages of PRM^fSAD^ (yellow text) and PRM^HAA^ (red text) are provided for additional context

To further assess FDSV subjects, a QCT-based radiographic phenotype of DRCB was derived using DRCB and Control subjects and used to create a DRCB-Probability Index (DRCB-PI). The model was created using demographic and adjusted PRM values obtained from DRCB and Control cohorts and captures the non-linear relationship between all adjusted PRM metrics to generate a probability value for each case ranging from 0 (least like DRCB) to 1 (most like DRCB). The findings of the model are displayed on a plot of adjusted PRM^fSAD^ and PRM^HAA^ as these metrics were strong determinants of overlap between DRCB and FDSV cohorts in our PCA (Fig. 2). This model achieved an area under the curve value of 0.91 in performance to delineate biopsy-proven DRCB from asymptomatic cases (Fig. 3A). Applying this model to our FDSV cohort (Fig. 3B) reveals subjects displaying an array of adjusted PRM^fSAD^ and PRM^HAA^ values. Applying a cutoff of > 0.5, we identified 10 out of 71 FDSV subjects as having a PRM phenotype that most resembled the biopsy-proven DRCB cohort (DRCB-PI > 0.5). Provided in Fig. 4 are two representative cases with low and high DRCB-PI. The case with a low DRCB-PI (of 0.05) is a 34-yr old female with moderate exposure to burn pit smoke during deployment. Values for PRM^fSAD^ (3%) and PRM^HAA^ (5%) were both low. In contrast, the case with a high DRCB-PI (of 0.93) is a 44-yr old male with severe exposure to burn pit smoke and values for PRM^fSAD^ (12%) and PRM^HAA^ (38%) were both high. Both cases were found to have normal values in FEV1 and FEV1/FVC.

Having established a means of distinguishing FDSV subject by QCT, we next sought to assess whether these subjects with a high DRCB-PI differed from those with a low DRCB-PI using other parameters. Comparisons between FDSV subjects with a DRCB-PI > 0.5 (*n* = 10) versus DRCB-PI ≤ 0.5 (*n* = 61) revealed similar demographics and traditional lung function measurements including spirometry, lung volumes, and gas exchange (Table 3). Respiratory oscillometry, however, differed between groups such that subjects with a DRCB-PI > 0.5 demonstrated greater frequency dependence of resistance (R5-R20; *P* = 0.001) and reactance area (AX; *P* < 0.01). Total resistance (R5; *p* = 0.055) and reactance (X5; *p* = 0.20) were also increased in subjects with DRCB-PI > 0.5, albeit non-significant. A DRCB-PI > 0.5 was also associated with airway wall thickening (elevated Pi10; *P* < 0.05) and impaired total lung ventilation (Jacobian Mean closer to 1.0; *P* < 0.001) in addition to the expected differences in PRM metrics (i.e. lower PRM^NORM^ and higher PRM^fSAD^ and PRM^HAA^) resultant from the model used to generate the DRCB-PI.

**Table 3:** Stratification of demographic, pulmonary function, and QCT metrics in FDSV subjects by DRCB probability index (DRCB-PI)

	DRCB-PI ≤ 0.5	DRCB-PI > 0.5	*p*
**N**	61	10	
**Age (yrs)**	45 (36, 52)	48 (39, 54)	0.5
**Sex (Male)**	51 (84%)	10 (100%)	0.3
**BMI (Kg/m** ^**2**^ **)**	31.7 (29.1, 36.2)	32.5 (30.1, 35.0)	> 0.9
**Ever-smoker (Y)**	25 (41%)	5 (50%)	0.7
**Pack Years (yrs)**	0 (0, 6)	2 (0, 7)	0.4
Unavailable	2	0	
**FEV1pp (%)**	96 (84, 108)	92 (74, 96)	0.3
Unavailable	2	2	
**FEV1/FVC (%)**	78 (75, 82)	78 (64, 81)	0.5
Unavailable	2	2	
**TLCpp (%)**	88 (80, 97)	92 (79, 101)	0.7
Unavailable	3	2	
**RV/TLC (%)**	23 (19, 30)	26 (15, 42)	0.6
Unavailable	3	2	
**DLCOpp (%)**	89 (79, 96)	85 (75, 102)	0.8
Unavailable	4	2	
**R5 (pp)**	98 (75, 133)	162 (112, 223)	0.055
Unavailable	22	2	
**X5 (pp)**	95 (49, 131)	153 (79, 391)	0.2
Unavailable	22	2	
**R5-R20 (kPa/L/s)**	0.41 (-0.01, 0.91)	2.17 (1.26, 3.50)	0.001
Unavailable	22	2	
**AX (kPa/L)**	9 (4, 17)	35 (26, 64)	0.002
Unavailable	22	2	
**Wall Area (%)**	45.8 (41.0, 48.4)	49.6 (46.7, 51.3)	0.12
Unavailable	8	2	
**Pi10 (cm)**	0.19 (0.14, 0.23)	0.29 (0.22, 0.31)	0.023
Unavailable	8	2	
**Jac Mean**	1.73 (1.59, 2.15)	1.35 (1.24, 1.46)	< 0.001
Unavailable	8	2	
**PRM** ^**Norm**^	54 (46, 65)	36 (27, 39)	< 0.001
**PRM** ^**fSAD**^	5.0 (2.2, 7.8)	11.5 (8.1, 12.0)	< 0.001
**PRM** ^**HAA**^	17 (8, 27)	29 (27, 46)	0.005
**PRM** ^**Emph**^	1.07 (0.06, 2.02)	1.79 (1.16, 2.20)	0.2

Data are shown as median (lower quartile, upper quartile), or N (%)

Lastly, the DRCB-PI was used to assess whether the QCT-derived DRCB phenotype distinguished FDSV subjects based on symptoms or self-reported health effects from deployment exposures (Table 4). Symptoms of cough, wheeze and SOB were common in all FDSV subjects yet increased in FDSV subjects with a DRCB-PI > 0.5 (relative to those with a DRCB-PI ≤ 0.5) although these findings did not reach statistical significance. Exposures to burn pit smoke, sand and dust, and petrochemicals were common in all FDSV subjects. Yet subjects with a DRCB-PI > 0.5 self-reported more severe health effects from exposures to burn pit smoke (*P* < 0.05) and sand and dust (*P* < 0.05) and a non-significant increase in severe petrochemical exposure (*P* = 0.063).

**Table 4:** Stratification of symptom and exposure metrics in FDSV subjects by DRCB probability index (DRCB-PI)

	DRCB-PI ≤ 0.5	DRCB-PI > 0.5	*p*
**N**	61	10	
**Cough**	29 (57%)	8 (80%)	0.3
Unavailable	10	0	
**Wheeze**	29 (59%)	8 (80%)	0.3
Unavailable	12	0	
**SOB**	44 (81%)	9 (90%)	> 0.9
Unavailable	7	0	
**Burn Pit Smoke**			0.028
Mild/None	9 (18%)	1 (11%)	
Moderate	29 (59%)	2 (22%)	
Severe	11 (22%)	6 (67%)	
Unavailable	12	1	
**Sand and dust**			0.041
Mild/None	10 (19%)	1 (10%)	
Moderate	29 (54%)	2 (20%)	
Severe	15 (28%)	7 (70%)	
Unavailable	7	0	
**Petrochemicals**			0.063
Mild/None	20 (40%)	3 (43%)	
Moderate	24 (48%)	1 (14%)	
Severe	6 (12%)	3 (43%)	
Unavailable	11	3	

Data are shown as N (%). SOB is shortness of breath

## Discussion

The current study responded to two prominent challenges that have limited our understanding of DRCB and additional forms of chronic lung injury that may exist in a subset of individuals formerly deployed to post 9/11 conflicts in Iraq and Afghanistan. First, non-invasive tests including conventional pulmonary function tests and clinical interpretations of HRCT scans are insensitive for DRCB detection 8, 12. Second, concerns about the risks and benefits of performing surgical lung biopsy limit the ability to evaluate new testing approaches in patients with biopsy-confirmed disease. We responded to these challenges by deriving a QCT-based radiographic phenotype of DRCB using HRCT obtained in military personnel with biopsy-proven DRCB and used this phenotype to augment our assessment of non-biopsied veterans. Our findings demonstrated that FDSV with the DRCB phenotype (DRCB-PI > 0.5) have greater abnormalities on oscillometry, increased airway wall thickness and greater impairments in total lung ventilation (detected by QCT), despite similar pulmonary function on conventional assessments relative to subjects with a DRCB-PI ≤ 0.5. These subjects also self-reported more intense health effects at the time of their exposure to military burn pit smoke and sand and dust storms. Collectively, our results show the potential for a QCT-derived radiographic phenotype of DRCB to improve non-invasive assessments of FDSV.

We previously showed that HRCT obtained on military personnel at the time they underwent surgical lung biopsy are amendable to QCT analysis using parametric response mapping 18. Results demonstrated that the amount of %PRM^fSAD^ present in military personnel with biopsy-proven DRCB is increased relative to asymptomatic controls and patients with mild to moderate COPD 18. In the current study, we found that the median %PRM^fSAD^ was also increased in the FDSV cohort. Yet completion of a more comprehensive PRM-based assessment revealed considerable heterogeneity in the amount of PRM^Norm^, PRM^fSAD^, PRM^HAA^, and PRM^Emph^ in both the DRCB and FDSV cohorts. We believe PRM heterogeneity may reflect the varying forms of chronic lung injury, including but not limited to DRCB, reported in multiple studies 8, 9, 10, 11, 32. This degree of observed PRM heterogeneity suggests that development of a QCT-based approach to DRCB phenotyping should not rely on a single PRM metric such as fSAD. Rather it motivated development of a composite PRM-based phenotype that accounted for all 4 PRM metrics and potentially confounding demographic variables. Thus, we derived the DRCB-PI as a composite metric to convey the degree to which a HRCT was similar to the QCT-derived DRCB phenotype.

Application of the DRCB-PI to the FDSV cohort yielded a spectrum of patients along the DRCB-PI continuum. Due to limited sample size, as a proof of concept a binary threshold of > 0.5 was chosen to identify the FDSV subjects whose QCT-derived radiographic phenotype was most comparable to subjects with DRCB. We found that the DRCB-PI did not differentiate FDSV subjects on the basis of conventional pulmonary function tests. In contrast, we found that FDSV subjects with a DRCB-PI > 0.5 had more abnormal oscillometry including increases in reactance area and frequency dependence of resistance (R5-R20), with additional non-significant increases in total resistance and reactance. This pattern of findings is suggestive of small airways disease. These findings extend those of Butzko and colleagues who had previously shown that 75% of FDSV subjects with preserved spirometry had one or more FOT abnormalities 27. Similarly, a study by Hines and colleagues had previously shown that airflow obstruction was more readily detected by impulse oscillometry relative to spirometry in assessment of Gulf War veterans 33. Thus, our analysis adds to the mounting evidence that conventional PFTs can be normal despite histopathologic and radiographic evidence of DRCB and suggest that oscillometry might prove more informative, particularly among those with environmental and occupational exposure [see Kaminsky et al. 2022 for review 34].

Additional QCT data revealed that FDSV subjects with a DRCB-PI > 0.5 had increases in Pi10 (an indicator of airway wall thickness), reductions in Jacobian Mean Ventilation (an indication of impaired total lung ventilation), and a trend towards increased emphysema (by %PRM^Emph^). These findings are compatible with a study by Zell-Baron and colleagues 35 which identified an increase in Pi10 and a quantitative CT metric for emphysema in their analysis of CT scans obtained from subjects they defined as having deployment-related distal lung disease. In contrast to our findings, their study did not identify quantitative CT evidence of increased air trapping. A direct comparison of our findings with these studies is limited by differences in both the deployed and control cohorts and the technique used to quantify air trapping.

The inability to reliably identify DRCB non-invasively have hindered prior efforts to understand symptoms and exposures associated with the disorder. In this study, application of the DRCB-PI to a subset of FDSV subjects that completed symptoms and exposure questionnaires revealed that FDSV subjects whose DRCB-PI was > 0.5 experienced more severe self-reported health effects immediately following exposures to burn pit smoke and sand and dust. The data further showed that FDSV subjects with a DRCB-PI > 0.5 also reported greater exposure to petrochemicals and symptoms of cough and wheeze, albeit non-significant. These findings bolster a recent report linking chronic respiratory symptoms with military exposures to burn pits and vapors, gasses, dusts and fumes 5 and suggest that a QCT-derived phenotype of DRCB may aid future efforts seeking to identify the cause(s) of DRCB.

### Limitations

HRCT scans from the DRCB and FDSV cohorts were obtained for clinical purposes and were not prospectively acquired using a standardized protocol. Thus, although our PRM analysis accounts for differences in scan acquisition 36, 37, differences in scanner, scan protocol, and exhalation volumes might still have impacted PRM measurements. In addition, our assessment of subjects with biopsy-proven DRCB was limited to subjects with histopathologic evidence of CB. It’s unknown whether additional interstitial, airspace, or vascular abnormalities were present, as has been reported in some subjects. 9, 10, 35. Data pertaining to inhalational hazards relied on self-reported exposures subject to recall bias. Also, there is a paucity of large well-characterized cohorts of asymptomatic or formerly-deployed symptomatic subjects that have undergone HRCT imaging. As such, small sample sizes may have limited our ability to identify additional significant associations between the DRCB-PI and other phenotypic metrics. Lastly, our subjects were assessed at specialty referral centers and our findings may not be generalizable to veterans evaluated at local health care facilities.

## Conclusions

In this study we show that a QCT-derived radiographic phenotype of DRCB identified a subset of formerly deployed symptomatic veterans with abnormal oscillometry and more severe self-reported health effects following inhalational exposures to burn pit smoke and sand and dust storms during military deployment. Further efforts are warranted to incorporate QCT-based phenotyping into non-invasive strategies to detect constrictive bronchiolitis and other forms of chronic lung injury.

## Electronic supplementary material

Below is the link to the electronic supplementary material.

Supplementary Material 1

## Data Availability

No datasets were generated or analysed during the current study.

## Abbreviations

CB: Constrictive bronchiolitis
CT: Computed tomography
DRCB: Deployment-related constrictive bronchiolitis
DRCB-PI: Deployment related constrictive bronchiolitis probability index
FDSV: Formerly-deployed symptomatic veterans
HRCT: High resolution computed tomography
PCA: Principal component analysis
PFT: Pulmonary function test
PRM: Parametric response mapping
PRM^Emph^: PRM-Emphysema
PRM^fSAD^: PRM-functional small airways disease
PRM^HAA^: PRM-high attenuation area
PRM^Norm^: PRM-Normal lung
QCT: Quantitative computed tomography
