# Supplementary material for: Use of quantitative CT chest imaging to derive and assess a radiographic phenotype of deployment-related constrictive bronchiolitis
Source: Respir Res. 2025 May 21;26:195. doi: 10.1186/s12931-025-03269-8 (PMC12093805; doi:10.1186/s12931-025-03269-8)
Supplement: Supplementary file 1 — Supplementary Material 1 [file 12931_2025_3269_MOESM1_ESM.docx]

**Online Data Supplement**

**Title**

Use of Quantitative CT Chest Imaging to Derive and Assess a Radiographic Phenotype of Deployment-Related Constrictive Bronchiolitis

*Study Populations*

The cohort of military personnel with deployment-related constrictive bronchiolitis (DRCB; n=37) consisted of U.S. active-duty military personnel referred for clinical evaluation between 2007 and 2017 at the Vanderbilt University Medical Center due to persistent respiratory symptoms that developed following military deployments to countries in the Southwest Asia Theater of Military Operations (predominantly Iraq and Afghanistan). As part of their clinical evaluation, military personnel underwent PFT, HRCT, and surgical lung biopsy. All biopsies displayed histopathologic features of constrictive bronchiolitis ^1^; the presence or absence of additional features of chronic lung injury including chronic interstitial or pleural inflammation and fibrosis, or vascular smooth muscle hypertrophy were not systematically characterized.

The cohort of formerly deployed symptomatic veterans (FDSV; n=71) consisted of U.S. veterans referred for clinical evaluation between 2010 and 2020 at the New Jersey VA War Related Illness and Injury Study Center due to persistent respiratory symptoms that developed following military deployments to countries in the Southwest Asia Theater of Military Operations (predominantly Iraq and Afghanistan). As part of their clinical evaluation, FDSV completed a standardized intake packet questionnaire that assessed inhalational exposures during deployments as well as respiratory symptoms. All veterans underwent full pulmonary function testing (PFT), respiratory oscillometry, and high-resolution CT (HRCT) scanning ^2^.

The Control cohort (n=98) consisted of healthy civilians evaluated between 2009 and 2012 at the University Medical Center Groningen in the Netherlands as part of a clinical study (“NORM, A Study to Obtain Normal Values of Inflammatory Variables from Healthy Subjects”, NCT00848406) ^3^. Subjects were >40 years of age who did not smoke during the last year and had <1.0 pack years smoking history. Subjects underwent PFT and HRCT.

Cumulative demographic data and results of Pulmonary Function Tests for all cohorts are provided in Table 2 of the primary manuscript. Additional details pertaining to their assessments are provided below.

*Pulmonary Function Testing*

All subjects across each of the three clinical cohorts underwent complete pulmonary function testing (PFT), including spirometry (FEV_1_, FEV_1_/FVC), body plethysmography (TLC, RV/TLC), and diffusing capacity of carbon monoxide (DLCO) according to American Thoracic Society guidelines ^4-6^. To facilitate comparison, we reported all PFT parameters as a percent of predicted value, with the exception of FEV1/FVC and RV/TLC which are reported as a ratio, using the Global Lung Function Initiative (GLI) reference equations ^7-9^. For one of our cohorts (DRCB), certain PFT parameters (FEV1, TLC, DLCO) were only available as a percent predicted value using alternative reference equations ^10-12^. In these cases, we derived raw PFT values as other reference equation factors were available (e.g., age, sex, height) and subsequently recalculated percent predicted values using the GLI reference equations.

*Respiratory Oscillometry*

Veterans with FDSV performed respiratory oscillometry as previously described ^2^ in accordance with published standards ^13^ as part of a comprehensive clinical evaluation between 2013 – 2019. In brief, oscillometry data were acquired using commercially available equipment (i2M, PFT Suite/Omnia, Cosmed; Rome, Italy) using device-specific measurement parameters (pseudorandom signal type; 4 – 48 Hz; 2 Hz resolution; 8 sec acquisition). Oscillometry was performed prior to other pulmonary function tests. Testing was performed in a seated position, using a nose clip and antibacterial filter, with the participant providing their own cheek support after receiving verbal and visual instruction from a trained technician. A minimum of three technically acceptable maneuvers, with a coefficient of variation ≤ 10% for resistance at 5 Hz across trials, were used for analysis. Total resistance (R) and reactance (X) at 5 Hz, area under the reactance curve (AX), and the frequency dependence of resistance (R5-R20) were selected as our primary variables of interest. Parameters were calculated using the mean of whole breaths only.

*Image acquisition*

For all cohorts, volumetric inspiratory scans were obtained at full inhalation; expiratory scans were obtained at approximate residual volume. All CT scans were checked for Hounsfield unit (HU) drift and if necessary corrected based on aortic blood (50 HU) and central air (-1,000 HU) as previously described ^14^. Scanner type and acquisition protocol for each site are provided in Supplemental Table S1 (below).

*Image Processing*

The fully automated software YACTA (version 2.9.4.74) was utilized to segment the lungs from the thoracic cavity on both inspiratory and expiratory CT images, and to conduct all airway analyses on inspiratory CT images ^15,16^. The inspiration scan was then spatially aligned to the expiration scan such that both share the same spatial geometry using Elastix an open-source deformable image registration algorithm ^17,18^. User veriﬁcation and manual corrections were applied as necessary. This process allows the paired images to share the same geometric space, where each voxel, the smallest unit of volume in a three-dimensional image dataset, consists of HU values at inspiration and expiration. Scans deemed unsuitable for PRM analysis owing to motion artifact and misalignment were excluded.

*Voxel classiﬁcation*

Each voxel was classified based on a scheme of three predetermined thresholds as previously described [Supplementary e-Table 1 and as described in Galban et al ^19^]. These thresholds are used to classify individual voxels into one of four categories with the following color codes: normal parenchyma (green colored voxels), fSAD (yellow colored voxels), high attenuation area (purple colored voxels), and emphysema (red colored voxels). Whole-lung PRM measures were calculated by normalizing the sum of all voxels within a classiﬁcation by the total lung volume, which include all parenchymal voxels over the full range of HU. The nomenclature of these measures for normal lung parenchyma, fSAD, emphysema, and high attenuation area were PRM^Norm^, PRM^fSAD^, PRM^HAA^, and PRM^Emph^, respectively.

| Ventilation | Parameter | Control | DRCB | FDSV |
| --- | --- | --- | --- | --- |
|  | Scan Year (Range) | 2009-2012 | 2007-2017 | 2010-2020 |
|  | Vender | SIEMENS | Philips/GE | Philips/Toshiba |
|  |  |  |  |  |
| Inspiration | Recon Kernel | B30f | B | B/FC09 |
|  | Slice Thickness (mm) | 1 | 1-3 | 0.5-1 |
|  | Slice Number | 305-578 | 103-681 | 369-1317 |
|  | Slice Interval | Contiguous | Contiguous | Contiguous |
|  |  |  |  |  |
| Expiration | Recon Kernel | B30f | L | B/FC09 |
|  | Slice Thickness (mm) | 1 | 1.25 | 0.5-1 |
|  | Slice Number | 248-578 | 27-60 | 369-1317 |
|  | Slice Interval | Contiguous | Incremental | Contiguous |

E **–**Table 1: Scanner type and acquisition protocols

References:

1. King MS, Eisenberg R, Newman JH, et al. Constrictive bronchiolitis in soldiers returning from Iraq and Afghanistan. *N Engl J Med.* 2011;365(3):222-230.

2. Butzko RP, Sotolongo AM, Helmer DA, et al. Forced oscillation technique in veterans with preserved spirometry and chronic respiratory symptoms. *Respir Physiol Neurobiol.* 2019;260:8-16.

3. Nair GB, Galban CJ, Al-Katib S, et al. An assessment of the correlation between robust CT-derived ventilation and pulmonary function test in a cohort with no respiratory symptoms. *Br J Radiol.* 2021;94(1118):20201218.

4. Culver BH, Graham BL, Coates AL, et al. Recommendations for a Standardized Pulmonary Function Report. An Official American Thoracic Society Technical Statement. *Am J Respir Crit Care Med.* 2017;196(11):1463-1472.

5. Graham BL, Brusasco V, Burgos F, et al. 2017 ERS/ATS standards for single-breath carbon monoxide uptake in the lung. *Eur Respir J.* 2017;49(1).

6. Graham BL, Steenbruggen I, Miller MR, et al. Standardization of Spirometry 2019 Update. An Official American Thoracic Society and European Respiratory Society Technical Statement. *Am J Respir Crit Care Med.* 2019;200(8):e70-e88.

7. Hall GL, Filipow N, Ruppel G, et al. Official ERS technical standard: Global Lung Function Initiative reference values for static lung volumes in individuals of European ancestry. *Eur Respir J.* 2021;57(3).

8. Quanjer PH, Stanojevic S, Cole TJ, et al. Multi-ethnic reference values for spirometry for the 3-95-yr age range: the global lung function 2012 equations. *Eur Respir J.* 2012;40(6):1324-1343.

9. Stanojevic S, Graham BL, Cooper BG, et al. Official ERS technical standards: Global Lung Function Initiative reference values for the carbon monoxide transfer factor for Caucasians. *Eur Respir J.* 2017;50(3).

10. Crapo RO, Morris AH, Gardner RM. Reference spirometric values using techniques and equipment that meet ATS recommendations. *Am Rev Respir Dis.* 1981;123(6):659-664.

11. Macintyre N, Crapo RO, Viegi G, et al. Standardisation of the single-breath determination of carbon monoxide uptake in the lung. *Eur Respir J.* 2005;26(4):720-735.

12. Pellegrino R, Viegi G, Brusasco V, et al. Interpretative strategies for lung function tests. *Eur Respir J.* 2005;26(5):948-968.

13. King GG, Bates J, Berger KI, et al. Technical standards for respiratory oscillometry. *Eur Respir J.* 2020;55(2).

14. Stoel BC, Stolk J. Optimization and standardization of lung densitometry in the assessment of pulmonary emphysema. *Invest Radiol.* 2004;39(11):681-688.

15. Leutz-Schmidt P, Weinheimer O, Jobst BJ, et al. Influence of exposure parameters and iterative reconstruction on automatic airway segmentation and analysis on MDCT-An ex vivo phantom study. *PLoS One.* 2017;12(8):e0182268.

16. Lim HJ, Weinheimer O, Wielputz MO, et al. Fully Automated Pulmonary Lobar Segmentation: Influence of Different Prototype Software Programs onto Quantitative Evaluation of Chronic Obstructive Lung Disease. *PLoS One.* 2016;11(3):e0151498.

17. Klein S, Staring M, Murphy K, Viergever MA, Pluim JP. elastix: a toolbox for intensity-based medical image registration. *IEEE Trans Med Imaging.* 2010;29(1):196-205.

18. Shamonin DP, Bron EE, Lelieveldt BP, et al. Fast parallel image registration on CPU and GPU for diagnostic classification of Alzheimer's disease. *Front Neuroinform.* 2013;7:50.

19. Galban CJ, Boes JL, Bule M, et al. Parametric response mapping as an indicator of bronchiolitis obliterans syndrome after hematopoietic stem cell transplantation. *Biol Blood Marrow Transplant.* 2014;20(10):1592-1598.
